# The Association between Deliberate Self-Harm and School Bullying Victimization and the Mediating Effect of Depressive Symptoms and Self-Stigma: A Systematic Review

**DOI:** 10.1155/2018/4745791

**Published:** 2018-10-11

**Authors:** Maria N. K. Karanikola, Anne Lyberg, Anne-Lise Holm, Elisabeth Severinsson

**Affiliations:** ^1^Department of Nursing, School of Health Sciences, Cyprus University of Technology, 15, Vragadinou Street, 3041-Limassol, Cyprus; ^2^Centre for Women's, Family and Child Health, Department of Nursing and Health Sciences, Faculty of Health and Social Sciences, University of South-Eastern Norway, P.O. Box 235, N-3606 Kongsberg, Norway; ^3^Department of Health Science, Western Norway University of Applied Sciences, Bjørnsonsgt, 45, 5528 Haugesund, Norway; ^4^Centre for Women's, Family and Child Health, Department of Nursing and Health Sciences, Faculty of Health and Social Sciences, University of South-Eastern Norway, P.O. Box 235, N-3603 Kongsberg, Norway

## Abstract

**Background:**

Identifying deliberate self-harm in the young and its relationship with bullying victimization is an important public health issue.

**Methods:**

A systematic review was performed to explore evidence of the association between deliberate self-harm and school bullying victimization in young people, as well as the mediating effect of depressive symptoms and self-stigma on this association. An advanced search in the following electronic databases was conducted in January 2018: PubMed/Medline; CINAHL; PsycINFO; PsycARTICLES; Science Direct; Scopus, and Cochrane Library. Studies that fulfilled the inclusion criteria were further assessed for their methodological integrity. The Norwegian Knowledge Centre for Health Services tool was applied for cross-sectional studies and the Critical Appraisal Skills Programme instrument for the cohort studies. Only empirical quantitative studies published in the English language in peer reviewed journals during the last decade (2007-2018) aimed at exploring the association between deliberate self-harm and school bullying victimization in community-based schoolchildren with a mean age of under 20 years were included.

**Results:**

The reviewed cross-sectional and cohort studies (22) revealed a positive association between school bullying victimization and deliberate self-harm, including nonsuicidal self-injury, which remained statistically significant when controlled for the main confounders. The mediating role of depressive symptoms in the association between deliberate self-harm and school bullying victimization was confirmed. A dose-response effect was shown in the association between nonsuicidal self-injury and school bullying victimization, whilst the mediating effect of depressive symptoms needs to be further explored. No studies were found directly exploring the mediating effect of self-stigma in the association between deliberate self-harm and bullying victimization.

**Conclusion:**

Targeted interventions aimed at eliminating victimization behaviours within the school context are therefore proposed, as well as interventions to promote healthy parenting styles for the parents of schoolchildren. Moreover, school healthcare professionals should screen students involved in bullying for self-injury, and vice versa.

## 1. Introduction

The construct of deliberate self-harm predating the fifth edition of the Diagnostic and Statistical Manual of Mental Disorders (DSM-5) encompassed a broad spectrum of behaviours that could damage the body in every possible nonfatal way, regardless of intention to die [1]. Moreover, the term “deliberate self-harm (DSH)” is often used interchangeably with “self-harm” and “self-injury” [1]. In contrast, nonsuicidal self-injury (NSSI), recently included in the DSM-5, is defined as an act of intentional self-directed harm to the surface of one's body without* conscious* intention to die [2]. Suicidal behaviour, including suicidal thoughts and/or suicide attempts, covers actions aimed at deliberately ending one's own life [2].

Based on the above, deliberate self-harm may be viewed as a wide spectrum of behaviours, characterised by different levels of suicide intent and a variety of motives [2–4]. Some of the self-harming behaviours include ingestion of a substance in excess or a nondigestible object; jumping from a height; and self-cutting or self-burning [5]. The main groups of motives behind these actions may be revenge against others and manipulation, intention to die, seeking attention, or escape from intolerable and agonising conditions [3]. Thus, the intention of deliberate self-harm ranges from no intention to end one's own life (NSSI) to strong intention to end one's own life (suicidal attempt/completed suicide), while it is also possible for multiple motives to coexist, for example, wanting to send a message and at the same time to obtain relief from suffering [2].

Deliberate self-harm constitutes a major public health issue, as its prevalence is increasing worldwide among young people [5–8]. Lifetime prevalence has been reported as 13.5% for females and 4.3% for males [5, 9–11]. Additionally, deliberate self-harm is one of the strongest predictors of suicide in the young [2, 12–16].

The presence of mental and behavioural problems has been linked with engagement in deliberate self-harm [2, 17–23]. Additionally, increased prevalence of deliberate self-harm as a socially deviant behaviour has also been identified in nonclinical populations [24]. Among others, dysfunctional relations with peers, peer rejection, bullying, and victimization have been associated with deliberate self-harm in the young [25]. Bullying victimization is defined as the experience of repetitive, aggressive behaviour towards an individual by her/his peers, such as unprovoked attacks, rejection and social isolation, humiliation and ridicule, malicious rumours, and name-calling, leading to severe distress in the victim, whilst the victim is unable to defend him/herself [26]. Bullying constitutes a public health problem, as approximately 32% of schoolchildren across 38 countries report experiences of peer victimization and subsequent emotional distress, whilst 10% of them may develop self-harming behaviour [6]. Different types of bullying victimization have been identified, such as verbal, relational, physical, or cyber, while the most common environment in which it takes place is school [1].

Bullying victimization during childhood or adolescence is a risk factor of poor physical health, mental health disorders, deliberate self-harm, and suicidal symptoms at any age [16, 27–31]. Bullying victims experience severe emotional distress associated with the psychological and physical violence they are subjected to, as well as social marginalization and decreased status among peers [27, 32–34]. A negative self-concept and decreased self-esteem, combined with perceived weakness and rejection by peers, have been described [35]. This process may be linked with the development of self-stigma. Developmental psychology supports that maltreatment and victimization during childhood and adolescence may be a key factor associated with self-stigma [36]. Self-stigma takes place when individuals internalize the negative public attitudes and stereotypes about their status and further experience a wide range of adverse costs related to these prejudices [36]. Thus, self-stigma evokes intense feelings of embarrassment, worthlessness, and self-blame influencing one's self-esteem. Decreased self-esteem, associated with self-stigma, has been associated with both deliberate self-harm and depressive symptoms [37].

Furthermore, self-stigma among those living with mental problems is also reported [38–41]. Data show that adolescents who present symptoms of borderline personality disorder, depression, psychosis, and conduct disorder are more likely to experience self-stigma [18, 40–43]. Additionally, these groups more frequently report both school bullying victimization and self-harming behaviour [44]. Based on this, one may hypothesise that bullying victimization, self-stigma, and deliberate self-harm may be present simultaneously. However, the link among these variables has not been explored adequately, either in clinical or in nonclinical populations [45, 46]. For instance, a cross-sectional study in 224 adolescents aged 13–17 years in rural Uganda provided evidence on the link among self-stigma, bullying victimization, severe depressive symptoms, and suicidality [45]. However, this study took place in adolescents with HIV; thus further investigation in the general population is needed. Additionally, other types of self-harming behaviours were not included in this study.

Nevertheless, preexistence of mental health and behavioural problems constitutes a risk factor for peer rejection and bullying victimization, mainly due to the social stigma related to mental illness [42–44]. At the same time, bullying victimization may have an additional negative influence on the clinical outcome of the symptoms experienced by those living with mental health problems or who have behavioural problems [2, 47]. Thus, the causality between bullying and mental health problems, such as depressive symptoms, self-harming behaviour, low self-esteem, and negative self-concept, seems to be bidirectional [43, 48, 49]. Consequently, it would be interesting to explore the mediating effect not only of depressive symptoms in the association between self-harming behaviours and bullying victimization but also of “mental health problem self-stigma” and/or “bullying rejection self-stigma” [34, 37, 40, 41, 50]. For instance, the study by Pantelic et al. [46] revealed the complex nature of self-stigma development, suggesting the existence of multilevel mechanisms prominent to it. In particular, Pantelic et al. [46] found that although self-stigma was associated with depressive symptoms, bullying victimization was not directly associated with internalized stigma, implying multiple risk pathways from personal problems to psychological distress; violence within homes, communities, and schools; and self- stigma development.

Although several studies have explored the association between bullying victimization and deliberate self-harm or suicidality [28, 51–55], only a limited number of them have systematically reviewed the articles exploring these associations [28, 51, 52]. In particular, two focused specifically on suicidal behaviour [28, 51] and one solely on NSSI. However, better understanding of the complexity of the association between deliberate self-harm and bullying victimization is needed, since an overlap between all types of self-harming behaviour and motives, especially in the young, has been reported [3]. Moreover, further research is needed regarding possible mediators in the association between deliberate self-harm and bullying victimization [28, 51, 52]. Comprehensive knowledge of the factors mediating the association of bullying victimization with self-harming behaviour in the young is crucial to further inform current health policies, preventive screening, and educational strategies, as well as developing targeted treatment programmes [56]. Thus, it is important to further explore and better understand the correlates of self-harming behaviours and their outcomes [4, 57]. The present review aims to present data regarding potential difference between those who engage in deliberate self-harm with and without clearly reporting intention to die, in relation to school bullying exposure and possible mediators, that is, depressive symptoms and self-stigma. Possible differences may further inform the phenomenology of these two self-harming groups.

The theoretical framework underpinning the present review is derived from the social-ecological theory [58]. Such a framework supports the hypothesis that a link exists between the school environment, risk of involvement in bullying, and the adverse impact of bullying on students, namely, self-harming behaviour, while highlighting the importance of related factors, such as depressive symptoms and self-stigma [58].

For the purpose of the present review, the term “deliberate self-harm” defines every nonfatal act performed intentionally by an individual with the aim of causing physical or psychological harm to her/himself, irrespective of suicidal intent [5]. As a result, the terms self- injury and suicidal behaviour are not used interchangeably in the present study [2]. Additionally, for the purpose of the present study self-stigma is defined as the situation in which a person believes the negative stereotypes related to her/his mental health problems and/or accepts the negative attitudes related to her/his bullying victimization and rejection [41].

School bullying victimization is defined as victimization of any type, that is, verbal, physical, and relational victimization within the school environment or among students [59].

## 2. Materials and Methods

### 2.1. Aim

The aim of the present systematic review was to assess the evidence of the association between deliberate self-harm, nonsuicidal self-injury, and school bullying victimization in young people, with special focus on the following research questions:

Is the effect size of the association between “deliberate self-harm and school bullying victimization” different from that of the association between “nonsuicidal self-injury and school bullying victimization”?

Is the association between deliberate self-harm, nonsuicidal self-injury, and school bullying victimization in the young mediated by depressive symptoms?

Is the association between deliberate self-harm, nonsuicidal self-injury, and school bullying victimization mediated by self-stigma?

### 2.2. Study Design

A systematic review of the literature was conducted. The main features of a systematic review comprise precisely reported research questions; implementation and description of a robust and reproducible methodology; a systematic search of scientific data in accordance with precisely stated criteria; assessment of the methodological quality of the reviewed studies; critical and systematic demonstration of the features; and main results of the reviewed studies [60, 61]. The above steps were applied in the present study.

### 2.3. Search Strategy

An advanced search in electronic databases was conducted between the 6th and the 25th of September, 2016, and was repeated in January 18th, 2018. A search in PubMed/Medline, Cumulative Index of Nursing and Allied Health Literature (CINAHL), PsycINFO, PsycARTICLES, Science Direct, Scopus, and Cochrane Library (clinical trials) was undertaken using the following keywords alone and in combination, in line with Medical Subject Headings: (self-harm or self-injury or deliberate or self-mutilation or self-abuse or self-injurious or suicidal or self-harming or suicide or “life-threatening behavior”) AND (depressive or depression or stress or self-harm or self-stigma) AND (bullying or victimization or “peer aggression” or intimidation) AND (young or youth or juvenile or adolescent or students). The term “self-harm” was used twice aiming to identify additional studies in which “self-harm” was studied as a mediator in the association between suicidality or self-threatening behaviour and bullying victimization, thus providing indirectly information about the association between self-harm and bullying victimization.

The search was conducted independently by two authors (MK and ES) and validated by a specialist librarian (KM). The latter independently rescreened to assess the rigour of the screening procedure.

### 2.4. Inclusion and Exclusion Criteria

The following inclusion criteria were set:

- Publication during the last decade (2007-2018) in the English, Greek, Norwegian, or Swedish languages and in a peer reviewed journal
- Empirical study design with any type of quantitative methodology
- Study sample of preadolescents or adolescents with a mean age of under 20 years
- Nonclinical population as the target group (i.e., the sample of each study had to be drawn from the community).
- Measurement of deliberate self-harm, nonsuicidal self-injury, and school bullying victimization by either a structured interview instrument, or a self-report scale, or a single question, clearly stated in the Methods section of the study
- Reported measures of the association between deliberate self-harm, nonsuicidal self-harm, and bullying victimization in the results of the studies
- Exploration of school-context bullying victimization, based on the wording of the measurement tool used for assessment of the bullying experience ( i.e., inclusion of the words “school” or “student”). An example is “how often were you left out of things, excluded, or ignored in school?” or “we can say a student is a victim of bullying when another student or a group of peers says malicious or hurtful things to him.”

**Figure 1:**
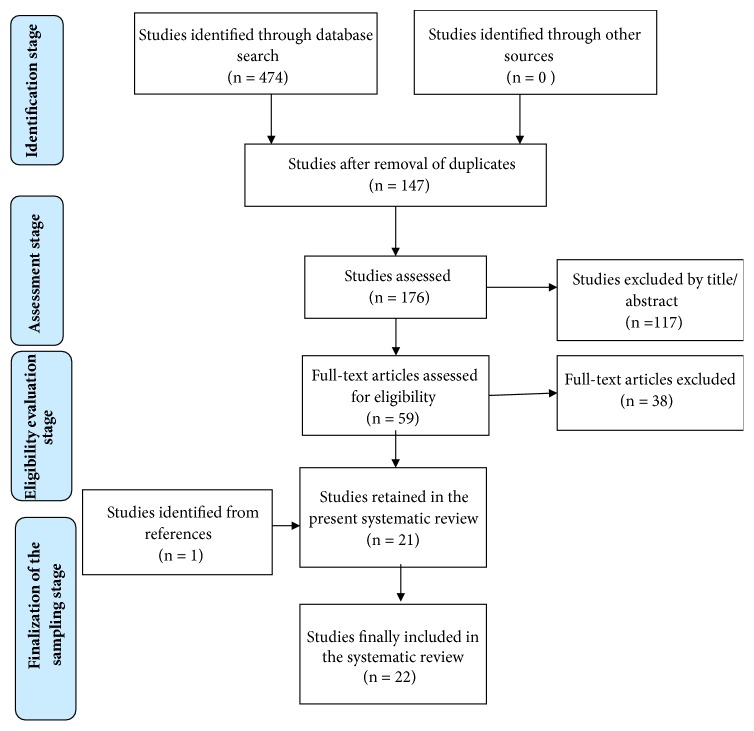
Selection strategy of the included studies based on the PRISMA flowchart [61].

The following studies were excluded after screening for eligibility (n=38) (Figure 1):

- Qualitative studies (n=2); theoretical studies (n=0); reviews, meta-analyses, and metasyntheses as systematic reviews focus on primary studies (n=3); protocols; educational studies and programmes (n=0); and monographs, guidelines, or national policy recommendations or guidelines, due to their limited cultural context audience (n=0)
- Studies with no direct measurement of the association between deliberate self-harm and school bullying victimization (n=3); studies which did not explain how the variables (self-harm/bullying) were measured or defined (n=4); studies in which the researchers did not provide information about the type of victimization experienced (n=3); studies regarding abuse (i.e., verbal, physical, or sexual abuse) (n= 2); studies conducted in a nonschool context (e.g., bullying among brothers and sisters) (n=1); and studies exploring attitudes on self-harm and bullying (n=1) or retrospective studies in adults (n=2), as well as studies on cyber self-harm (n=1)
- Studies exploring indirect experiences of suicidal behaviours or self-harm related behaviours, such as witnessing deliberate self-harming behaviours of others, viewing suicide-related material, underage motorbike riding, or alcohol consumption (n=3); the reason behind this was that these studies lack direct assessment of self-harm. Additionally, studies investigating the association between school bullying victimization and direct suicidal behaviour, whilst not including deliberate self-harming, were also excluded (n=3).
- Studies in vulnerable populations, including prisoners and sex offenders (n=1); people with physical and/or mental illness comorbidities (n=1); homosexuals (n=1); and mental health clinical populations (e.g., bipolar patients or children with Attention Deficit Hyperactivity Disorder (n=3)), studies including only males or only females (n=2), or studies investigating the same sample as another study that was already included (n=2).

### 2.5. Selection Strategy

The selection strategy of the included studies was based on the PRISMA procedure [61] (Figure 1). The combined search identified 474 articles. After removing duplicates (298) and screening titles and abstracts for relevance to the aim and research questions of the review, 59 articles remained. Two researchers (MK and ES) independently screened both titles and abstracts of all retrieved articles for eligibility and resolved disagreements by consensus. One paper was added after a citation search; thus the full texts of 59 articles were studied thoroughly in relation to additional criteria pertaining methods, setting, and target population. Next, the full texts of the selected articles were screened for eligibility by all four researchers and any disagreements were resolved by consensus. When all the inclusion and exclusion criteria had been considered, 22 articles remained and comprised the sample of the present review. The 22 studies were coded for the variables explored herein.

Each included study was independently reviewed by two of the four researchers in accordance with the variables presented in Tables 1, 2, and 3, thus corresponding to the measures employed in the aims of the review, that is, context of the bullying (school/ students), target population (schoolchildren), methods (study design, definitions, measures, and tools employed), mediators in the association between school bullying victimization and deliberate self-harm (depressive symptoms and self-stigma), outcomes measured, and important results. A specially designed extraction sheet was used for data collection purposes, whilst the reason for excluding a study was documented.

### 2.6. Quality Assessment

The 22 studies that fulfilled the inclusion criteria were further assessed for their methodological integrity. The Norwegian Knowledge Centre for Health Services (NOKC) tool was used for assessing the methodological integrity of cross-sectional studies [62–64] and the Critical Appraisal Skills Programme (CASP) instrument for cohort studies [65]. Both tools provide an outline for assessment and critical appraisal of risk of bias related to confounding factors, participant selection, measurement, and data analysis. Based on a literature review on the subject of confounders, we identified age, gender, suicidal behaviour/previous suicide attempts, substance abuse, depressive and anxiety symptoms, self-esteem/self-concept, and impulsivity as critical confounders for inclusion to ensure the relevance of the study and reduce the risk of bias.

As the focus was on the association between deliberate self-harm and exposure to school bullying victimization, the NOKC checklist for cross-sectional studies was applied for analytic cross-sectional comparative and noncomparative studies. In cases where two or more groups of students were compared with regard to exposure to school bullying victimization and its association with deliberate self-harm, additional criteria adopted from the NOKC checklist for cohort studies were applied, as previously reported in the literature [62, 63]. Moreover, one further criterion regarding the ethical integrity of the included studies has been also added herein [64]. Thus, a modified version of the NOKC assessment tool including 13 questions has been used, addressing both comparative and noncomparative cross-sectional analytic study designs. These 13 NOKC checklist criteria are reflected in the following questions. (1): Is the target population of the study clearly defined? (2): Is the sampling method appropriate and is the study sample representative of the target population? (3): Is the nonexposed group selected from the same population as the exposed group? (4): Are the nonexposed and exposed groups comparable regarding the main background variables? (5): Is the degree and way, in which the respondents who consented to participate differ from those who did not, described? (6): Is the response rate satisfactory? (7): Is the method for data collection consistent? (8): Are the measures of the main variable reliable and valid? (9): Are the methods of statistical analysis suitable? (10): Are both exposure and outcome measured consistently in both groups (exposure/ nonexposure)? (11): Is the assessment of the outcome blind to whether participants were exposed or not? (12): Are the main confounders included in the study design? (13): Are the ethical issues properly addressed by the researchers?

The assessment of the rigour of the cohort studies was based on the CASP tool [65] (CASP 2014). This tool includes 12 criteria, organized in three groups of questions. First Section* (Validity of Study Results):* Is the study addressing a clearly focused topic? Has the cohort been recruited in a valid way? Is the exposure to the risk factors precisely measured to eliminate bias? Is the study outcome precisely measured to eliminate bias? Are all important confounding factors identified in the study design and further included in the data analysis? Is the follow-up procedure comprehensive and long enough in duration? Second Section* (Study Results):* What are the results of the study? How accurate are the study results? Do you think that they are important? Third Section (*Implementation of the Results in the Study Population*): Can the results be implemented in the study population? Are the study results in accordance with other available data? What are the implications for clinical practice based on the study results?

Studies that met over 50% of NOKC or CASP criteria were deemed to be of moderate quality, while those that met 70% or more of the criteria were classified as high quality. Studies that met 50% or less of these criteria were categorised as low quality (Tables 3 and 4) [62].

### 2.7. Data Analysis

Data were analysed in four steps: firstly, all researchers corroborated the definitions of deliberate self-harm, school bullying victimization, and internalized stigma to be used herein, whilst potential theoretical frameworks linked with the aforementioned definitions were then discussed; the next step involved the identification of studies that fulfilled the inclusion criteria and the description of their main methodological characteristics and relevant tables, as well as the assessment of their methodological quality; the final stage encompassed the organization of the results of the included studies in relation to the present research questions and the interpretation of data regarding the association between school bullying victimization and deliberate self-harm. Special focus was placed on the mediating effect of depressive symptoms and self-stigma and on the differences between deliberate self-harm and nonsuicidal self-injury behaviour, in accordance with the research questions.

## 3. Results

### 3.1. Methodological Characteristics, Definitions, and Measurements in the Reviewed Studies

A total of 205,805 community-based schoolchildren with a mean age of approximately 14.5 years (minimum mean age=12.3 years and maximum mean age=16.67 years) were assessed in the included studies. Tables 1 and 3 present the methodological characteristics of the 22 included studies. The range of the duration of follow-up in the cohort studies was 5 months to 18 years, including both birth cohorts and population-based community cohorts. 

Ten out of the twenty-two studies reviewed used a definition that concretely described NSSI [66–75]. The remaining twelve studies did not focus on intention to die [76–87]. The definitions and measurements of the main variables of the reviewed studies are presented in Table 2. Twelve studies assessed lifetime prevalence of deliberate self-harm and nonsuicidal self-injury, while the others explored these experiences during the three to 12 months previous to the study [66, 68, 71, 73–78, 87].

### 3.2. Is the Effect Size of the “Deliberate Self-Harm and School Bullying Victimization” Association Different from That of the “Nonsuicidal Self-Injury and School Bullying Victimization?”

#### 3.2.1. Prospective Association between NSSI and School Bullying Victimization

The prospective studies confirmed an association between exposure to school bullying victimization and lifetime prevalence of NSSI [72], as well as occurrence of NSSI in the past 3-12 months [68, 72, 73]. The effect size [OR (95% CI)] ranged between 1.23(0.80-1.89) and 2.19 (1.42-3.39). The highest score regarded NSSI reported in the previous year and by the victims themselves [73]. Moreover, the severity of exposure to school bullying victimization, either verbal or physical bullying, predicted the frequency of NSSI, confirming a prospective, dose-response association. These data were extracted from studies identified as being of high methodological quality [62].

#### 3.2.2. Cross-Sectional Association between NSSI and School Bullying Victimization

All cross-sectional studies supported a positive association between school bullying victimization and NSSI [67, 69], even after controlling for sociodemographic covariates (i.e., gender, age, ethnicity, and parental education) [70, 74, 75]. The effect size [OR (95%CI)] of this association ranged between 1.33 (0.67-2.64) and 4.75 (2.36-9.54) for occasional school bullying victimization, while the effect size was even stronger for repetitive school bullying victimization [11.75 (5.54-24.94)], demonstrating that even occasional school bullying victimization was associated with increased risk for NSSI [71, 74, 75, 77]. These scores regard NSSI incidents reported in the previous year [71, 74]. The aforementioned data were extracted from studies identified as being mainly of moderate methodological quality [62].

#### 3.2.3. Prospective Association between Deliberate Self-Harm and School Bullying Victimization

Exposure to frequent school bullying victimization before the age of 12 years (i.e., 7 to 10 years) was associated with increased risk for deliberate self-harm at the age of 12 years and in late adolescence [82–84]. The effect size [OR (95% CI)] ranged between 1.70 (1.4-2.2) and 3.53 (1.91-5.82). These data were based on reports of both the child and the mother. Similar scores were noted in relation to lifetime prevalence of deliberate self-harm and data reported by school bullying victims themselves [the highest value was 3.33 (1.91-5.82)] [82]. Interestingly, higher risk values of deliberate self-harm (i.e., 4.57 (1.66-12.54)) were based on bullying victimization data reported by teachers [82].

#### 3.2.4. Cross-Sectional Association between Deliberate Self-Harm and School Bullying Victimization

Similar to the above, the cross-sectional studies revealed that peer victimization by schoolmates was more frequent among those engaging in deliberate self-harm of any frequency, both occasional and repetitive. The effect size [OR (95%)] ranged between 1.10 (1.08-1.13) and 3.09 (2.06-4.64) [78, 79, 81, 87]. The highest effect size was reported by female victims of school bullying. All the aforementioned values of deliberate self-harming incidents were reported in the previous year [81]. Similarly, lifetime prevalence values of deliberate self-harm ranged between 1.33 (1.18-1.50) and 2.83 (1.50-5.36) [43, 67]. These values were reported by the school bullying victims themselves. Moreover, the highest value was measured among boys, with the lowest among samples of both genders.

### 3.3. Is the Association between Deliberate Self-Harm, Nonsuicidal Self-Injury, and School Bullying Victimization Mediated by Depressive Symptoms?

Regarding the mediating role of depressive symptoms in the cross-sectional association between school bullying victimization and deliberate self-harm, Hay & Meldrum [66] found that depressive symptoms only partially mediated this relationship when controlled for age, gender, ethnicity/origin, impulsivity, type of parenting, family type, and school performance [66]. Similarly, Espelage & Holt [76] reported that deliberate self-harm was statistically significantly higher in school bullying victims compared to students uninvolved in bullying, while depressive symptoms only partially explain that difference. With regard to prospective studies, it was shown that exposure to frequent school bullying victimization before the age of 12 years was a risk factor for deliberate self-harming behaviours in preadolescence for those who also reported depressive symptoms [82]. Data also revealed that adolescents who reported at least one incidence of deliberate self-harming behaviour were more likely to report experiences of all types of bullying victimization, while depressive symptoms only partially mediated this relationship [72].

With regard to NSSI, the cross-sectional study by Claes et al. [69] confirmed that exposure to school bullying victimization predicted NSSI, which was partially mediated by depressive symptoms [69]. Heilbron & Prinstein [68] in their cohort study found no main effect of school bullying victimization on the prediction of NSSI when controlled for depressive symptoms. In contrast, Giletta et al. [73] found a prospective dose-response effect between the frequency of exposure to school bullying victimization and the severity of NSSI, independent of depressive symptoms. These studies were of high quality.

### 3.4. Is There a Mediating Effect of Self-Stigma on the Association between Self-Injury, Nonsuicidal Self-Injurious Behaviour, and School Bullying Victimization?

With regard to this research question, none of the included studies directly explored this association.

## 4. Discussion

All reviewed studies confirmed a positive association of deliberate self-harm and NSSI with school bullying victimization, even when controlled for the main confounders (school grade, gender, depressive symptoms, suicidal behaviour, impulsivity, substance use, family history, and abuse). The mediating role of depressive symptoms in the association between deliberate self-harm and school bullying victimization was confirmed [66, 69, 72, 76, 82]. In contrast, the role of depressive symptoms in the association between NSSI and school bullying victimization was equivocal. Three studies measured this relationship. While one revealed a prospective, dose-response effect independent of depressive symptoms, the other two, one cross-sectional and one cohort, identified depressive symptoms as a mediator in this association [68, 73]. None of the reviewed studies directly explored the mediating effect of self-stigma on the association of self-injury, NSSI, and school bullying victimization.

The strength of the present results stems from the fact that the reviewed studies were of satisfactory methodological quality (moderate and high quality). Despite this, a lack of consensus regarding the definitions used for deliberate self-harm in the studies reviewed was observed. This is also reflected in the variety of terms applied, which also do not differentiate between self-harm with or without intention to die. The distinction between NSSI and deliberate self-harm is challenging, highlighting the need for future studies to clarify differences in risk profiles [4, 57]. A clear definition of NSSI is proposed for future studies aiming to achieve consensus regarding study variables, thus facilitating data comparisons among research groups, and leading to more accurate comprehension of both the context and the outcome of relevant behaviour. However, the way an individual hurts her/himself does not always reflect the intention to die, evidence of which is clearly reported in the CASE study [5]. Thus, it is vital that future studies clearly address the motive(s) behind self-injurious behaviour and not only the means by which it is inflicted [4, 57]. Additionally, clinicians need to assess the motives leading to self-injury so that the focus and prioritisation of interventions can be modified accordingly. Yet, one may argue that those who engage in self-harming behaviours, both nonsuicidal and suicidal, report similar clinical features and high rates of mental disorder and the way these two categories of individuals choose to hurt themselves does not seem to distinguish one group from the other. Overall, both groups appear to present similarities in diagnostic and demographic variables, making it extremely challenging to draw conclusions about the differences in the suicide risk among them. Thus, further research on this topic is necessary.

Furthermore, the present findings are in line with the social-ecological theory [58], since the majority of the schoolchildren reviewed herein did not engage in adverse phenomena related to bullying victimization, that is, deliberate self-harm or NSSI [82]. Those who were involved in deliberate self-harm were found to be affected by several personal, interpersonal, and social factors, including depressive symptoms. Other risk factors in that case were (a) having a family member who attempted/completed suicide, (b) experience of being physically abused by an adult, and (c) conduct disorder, borderline personality characteristics, and depressive and psychotic symptoms [82]. A dose-response effect regarding nonrecurring incidents in the association between school bullying victimization and deliberate self-harm was not confirmed, since only repetitive school bullying victimization was associated with self-harming behaviour.

Furthermore, the results regarding the mediating effect of depressive symptoms on the association between NSSI and exposure to school bullying victimization were equivocal. A meta-analysis on this topic showed that bullying victims reported NSSI more frequently than uninvolved schoolchildren. However, this meta-analysis did not explore the mediating effect of depressive symptoms on this association [52]. In contrast, the mediating effect of age on the relation between NSSI and school bullying victimization was explored, presenting that younger responders reported higher effect sizes than older schoolchildren [52]. The same meta-analysis also found that the association between NSSI and bullying victimization was not moderated by the way the respondents were selected (i.e., randomly or not); the response rate in the reviewed studies; the country in which the studies took place (i.e., the USA or a European country). These issues may be of relative importance for future study designs.

Overall, studies using the definition of deliberate self-harm without discriminating between suicidal and nonsuicidal intention reported a higher effect size of school bullying victimization compared to studies exploring NSSI. One possible explanation might be the fact that the use of a broad definition of deliberate self-harm does not allow distinction between the different types of motive for such behaviour, thus ignoring several types of psychopathology behind this conduct, such as mood disorders. In contrast, a strict definition providing a clear motive behind the self-injury (i.e., relief from distress) probably excludes cases in which mood disorder symptoms may prevail.

The studies reviewed herein did not directly assess the mediating effect of self-stigma; however, factors indirectly associated with it, such as low self-esteem or sexual and physical abuse, were included in the reviewed studies. The study by Lereya et al. [59] showed that there is an indirect association between exposure to maladaptive parenting and domestic violence before the age of 4 years and self-injuring behavior at the age of 16-17 mediated by school bullying victimization reported at the age of 7,8, and 10. According to these findings [59] one may argue that maladaptive behaviour in adolescents due to a dysfunctional family environment and poor parenting may possibly attract negative peer attention and peer rejection, leading to self-stigma related to social marginalization from peers [42, 44, 59, 88]. At the same time, the study by Heilbron and Prinstein [68] revealed that those adolescents who were less accepted by their peers and had low popularity in terms of both reputation and preference were more likely to be targets of overt and relational bullying victimization and to engage in self-injurious behaviour. This may also imply that social marginalization and consequent self-stigma may be factors involved in the association between school bullying victimization and self-harm. However, the mediating effect of internalized self-stigma due to social marginalization and low peer status was not tested in the group of adolescents who were targets of school bullying victimization and at the same time had reported self-injurious behavior in this study [68].

Further research aiming to explore the association of self-stigma from any cause with both self-injurious behaviour and school bullying victimization may be proposed. Thus, a different perspective is needed in the way possible mediating factors are studied in the association between deliberate self-harm and bullying involvement as well as new etiological links. This is really important in view of the fact that self-harming behaviour is a spectrum rather than a continuum of behaviours for young people, whilst even severe suicide attempts may arise with no warning signs or risk factors as yet identified in the literature. Thus, in order to develop interventions that target specific risk factors it is necessary to identify the etiological pathways from the experience of risk factors during childhood to the development of self-injurious behaviour during adolescence and adulthood. Overall, relevant interventions should focus on educating children and adolescents on how to effectively cope with stressors, including bullying, and how to maintain high self-esteem [59, 80, 81, 86], along with preventive and support policy in community, school environment, and healthcare system [46, 88].

## 5. Conclusions

School bullying victimization is a risk factor for deliberate self-harm, including both suicidal and nonsuicidal acts. Targeted school-oriented interventions at organizational level aimed at eliminating victimization behaviours within the school context are proposed, as well as community-oriented interventions focused on healthy parenting style and ways of coping with stress. The Saving and Empowering Young Lives in Europe study includes data which show that school-based preventive programmes (i.e., mental health educational interventions for pupils) are beneficial in preventing self-harming behaviours [89]. Moreover, as this review revealed a prospective association between exposure to school bullying victimization and deliberate self-harm in the young, school healthcare professionals (i.e., school nurses or educational psychologists) need to screen students involved in bullying for self-injurious behaviour and vice versa.

Psychological and psychosocial interventions may be effective in treating schoolchildren who already engage in self-harming behaviours [56, 90]. Cognitive behavioural therapy or dialectical behavioural therapy has been shown to be effective in adults [91]. Thus, further research in children and adolescents may be warranted. Additionally, in cases of limited availability of mainstream psychiatric services interventions from sources other than healthcare professionals (e.g., postcards or online support services) may provide support and relief from distress [90]. However, such interventions need to be investigated in future trials in children and adolescents [92]. Additionally, further longitudinal studies on new possible mediating factors in the association between deliberate self-harm and bullying involvement may provide further data regarding self-harming process in the young.

## 6. Limitations

A main limitation of the present study is that different definitions and instruments of deliberate self- harm were used in the studies reviewed. This inconsistency makes it difficult to identify the real extent of the phenomenon across different cultural contexts and compare data. Consequently, an effort was made herein to use a comprehensive, broad definition of deliberate self-harm and further differentiate it from NSSI during the analysis and interpretation of the data [1, 93]. Another limitation is that samples of individuals who were both bullies and victims of bullying were not included herein. Despite these limitations, the validity of this review is ensured by a comprehensive literature search, discussions about the search words, and inclusion criteria, as well as assessment of the risk of bias of the reviewed studies by means of the modified CASP and NOKC scores. Furthermore, although these tools provide a thorough assessment of the majority of bias risks, it is worth noting that bias related to missing data and publication bias are not included. Additionally, although we took into consideration the most frequently reported confounders, additional factors exist, such as coexistence of other types of victimization, maltreatment, type of parenting, social support, stressful life events, and interpersonal stress. Although documented, these were not identified among the most important. Moreover, confirmation of the data extracted herein by the primary investigators of the reviewed studies was not performed. However, all sources provided within main texts, additional files, and supplements of the published studies were screened.

## Figures and Tables

**Table 1:** Main results of the included studies pertaining to the research questions in the present systematic review.

**Authors & Date (study acronym)**	**Aim**	**Study Population**	**Main Results**
Brunner et al. 2014 (SEYLE study)	Exploration of the prevalence and associated psychosocial factors of occasional and repetitive direct self-injurious behaviour.	Community-based adolescents (age range: NR); n= 12,068; RR: 49%.	SBV & SB were both found to be strong predictors of SI in the univariate regression analysis (UA) and also showed an independent effect on the multivariate regression analysis (MA); SBV as a risk factor for lifetime SI: UA/ OR (95% CI): 3.39 (2.96-3.89) & MA/ 1.68 (1.43-1.98), p<0.01

Brunstein Klomek et al. 2016 (SEYLE study)	To examine the association between victimization by bullying and direct self-injurious behaviour.	Community-based adolescents (age range: NR); n= 11.110; RR: 72% of the schools approached.	SBV(physical/verbal/relational) was strong predictors of SI (lifetime, both occasional and repetitive) in the univariate regression analysis and also showed an independent effect on the multivariate regression (MA) model: SBV (physical/verbal/relational) as a risk factor for lifetime SI: UA/ OR (95% CI): 2.10-2.78 (1.90 to2.51 – 2.32 to 3.08) & MA/ 1.33-1.72 (1.18 to 1.49-1.50-2.07), p<0.01 The effect of relational & verbal SBV on SI was partially mediated by depressive symptoms (Sobel test = 7.33; p<0.0001; Sobel test=5.22; p<0.0001).

Claes et al. 2015	Investigation of the association between bullying victimization and NSSI and the mediating effect of depressive symptoms & parental support.	Community-based adolescents 7^th^- 12^th^ grade; n= 785; RR: NR	The association between SBV and NSSI (*β*=0.23, p<0.001) was partially mediated by depressive symptoms [x^2^ = 16.44, p=0.003; CFI=0.948; RMSEA=0.063].

Elgar et al. 2014 (2012 Dane County Youth Assessment Study)	Exploration of the association among cyberbullying victimization, traditional bullying, self-injurious behaviour & related mental health problems, as well as the moderating role of family contact.	Community-based adolescents 12-18 years; n= 18.834; RR: 90%.	SBV was a risk factor for SI [OR (95% CI): 1.10 (1.08- 1.13)].

Espelage & Holt 2013	Exploration of the differences in the frequency of suicidal ideation and suicidal behaviours across a group of verbal bullies, bully victims, victims, physically aggressive bullies, and students not involved in bullying.	Community-based adolescents 5^th^- 8^th^ grade (10-13 years; n= 661; RR: 93%.	SI was statistically significantly higher in victims of school bullying compared to uninvolved students (28.2% vs. 8.7%; x^2^ = 53.89; p<0.001), while depressive symptoms only partially explain the difference (F= 126.5; p<0.0001; n^2^ =0.17).

Ford et al. 2017 (K-cohort Longitudinal Study of Australian Children)	Exploration of the association between all types of bullying and adverse mental health outcomes, including self-harming behaviours and suicidality.	Community-based adolescents 14-15 years; n=2304; RR:59%	The prevalence (%)(95%CI)]) of self-harm in adolescents of both genders self-reported as bullying victims (all types of bullying victimization) was higher [18.6 (15.1-22.7)]compared to uninvolved adolescents [5.3(4.3-6.5)], adjusted score. Similarly, the risk factor for self-harming behaviour was higher in bullying victims [3.4(2.4-4.7)], adjusted score (gender, household type & income, home language spoken, parental education, Aboriginal/Torres Strait Islander heritage).The highest risk factors regarded experience of all three types of SBV [OR(95%CI): 4.6 (3.2-6.6)] and the lowest physical SBV[OR(95%CI): 2.9 (1.7-4.8)]. The mediating effect of DS in the association between SBV and SI was not assessed.

Garish & Wilson 2010	Investigation of potential risk factors for deliberate self-harm among adolescents, specifically focusing on peer victimization and alexithymia, as well as the mediating effect of depressive symptoms.	Community-based adolescents 16-23 years; n= 325; RR: 86%	Adolescents who reported at least one incidence of SI were more likely to report experiences of all types of SBV (*df>302, *t> 3.30, p<0.005). A positive association was also reported between SBV and SI (0.36, p<0.001). Depressive symptoms only partially mediated the relationship between SBV and SI, as the association between SBV and SI decreased (b weigh from 0.36 to 0.25) after controlling for depressive symptoms.

Giletta et al. 2012	Exploration of risk factors for NSSI including bullying victimization, in adolescents across three different countries (Italy, the Netherlands, and the USA).	Community-based adolescents 14-19 years; n= 1,862; RR:79.7%(n=82) in Italy; 89.3 % (n=675) in the Netherlands; and 50.6% (n=360) in the USA.	In multivariate analysis SBV was a risk factor for NSSI for the entire sample: sociodemographic covariates adjusted score (gender, age, ethnicity, and parental education) OR (95% CI): 1.96 (1.50-2.57), p<0.0001. In the subgroups: Italy: OR95%CI: 1.61 (1.08-2.41), p<0.05 The Netherlands: OR(95%CI) ]: 2.80(1.74-4.50), p<0.0001 USA: OR95%CI: 1.33(0.67-2.64), p<0.0001

Gower & Borowsky, 2013 (2010 Minnesota Student Survey)	Exploration of the association between the frequency of bullying involvement and both internalizing and externalizing problems, including deliberate self-harm.	Community-based adolescents 6^th^-12^th^ grade; n= 128.681; RR: 71%	Infrequent (1-2 times in ones' lifetime compared to no SBV was a risk factor for engagement in SI (6^th^, 9^th^, and 12^th^ grade) SI boys OR(95% CI): 1.93 to 2.17 (1.71 to 1.92 – 2.17 to 2.44), p<0.001 SI girls OR(95% CI): 2.07 to 2.23 (1.91 to 2.00 – 2.23 to 2.47), p<0.001 Even occasional SBV is associated with SI during adolescence.

Hay & Meldrum, 2010	Exploration of the hypotheses that bullying victimization is significantly related to NSSI & SI, mediated by DS.	Community- based adolescents 10-21 years; n=424; RR: 93%	NSSI was associated with SBV (B=0.32, p<0.001/ Adjust R^2^ =0.23), partially mediated by depressive symptoms (B=0.18, p<0.001/ Adjust R^2^ =0.28) controlled for age, gender, ethnicity/origin, impulsivity, type of parenting, family type, and school performance. [reduction of 44% in the association]

Jantzer et al. 2015	Exploration of the relationship between school bullying victimization (Type/Frequency) and NSSI/ SB.	Community-based adolescents 9-18 years; n= 647; RR:NM	Repetitive SBV was a risk factor for NSSI [NSSI OR(95% CI): 11.75(5.54-24.94), p<0.001]; Occasional SBV was a risk factor for NSSI [NSSI OR(95% CI): 4.74 (2.36-9.54), p<0.001]; There was a difference in the effect size between repetitive and occasional SBV: Variance explained regarding the prediction of SBV was R^2^=0.053 and for the prediction of NSSI R^2^=0.093 (no effect of grade/gender) => Dose-response relationship regarding the frequency of SBV [Even occasional SBV had an impact on SI]. The mediating effect of depressive symptoms was not assessed.

McMahon et al. 2010 (CASE study)	Exploration of the association between self-injury and psychological, life-style, and stressful life event- related factors in school adolescents.	Community-based adolescents 15-17 years; n= 3881; RR: 85%	SBV was a risk factor for a lifetime history of SI in both genders in univariate analysis: [girls OR: 2.61 95% CI (1.97-3.46), p<0.0005 / boys OR: 4.07 95% CI (2.57-6.44), p<0.0005]. In multivariate analysis to predict a lifetime history of SI, SBV remained a risk factor only for boys [OR age-adjusted score (95% CI): 2.83 (1.50-5.36), p=0.001]
			Although depressive symptoms were reported as a risk factor for SI in univariate analysis for both genders [OR 1.25-1.27; 95% (1.18-1.22 to 1.32; p<0.0005)], it was not the case in multivariate analysis, implying that when other variables such as SBV exist, the independent effect of depressive symptoms on SI is not significant in adolescents.

Noble et al. 2011/ Kansa**s** City/USA (Kauffman Teen Survey)	Exploration of the association between NSSI and perceived school safety & trust.	Community-based adolescents 11-19 years; n= 1276; RR: NM	SBV in a high school group was a risk factor for NSSI [B=0.41, SE:0.22, OR: 1.52, p<0.01]. SBV in a middle school group was a risk factor for NSSI [B=0.55, SE:0.18, OR: 1.73, p<0.01]. The mediating effect of depressive symptoms was not assessed.

O'Connor et al. 2009/ Scotland, UK (CASE Study)	Exploration of the prevalence of DSH and related factors in Scottish adolescents	Community-based adolescents 15-16 years; n= 2008; RR: 80%	SBV was found to constitute a risk factor for SI in both genders (The lifetime prevalence of SBV was strongly associated with SI in both boys and girls): [girls OR(95%CI): 3.09 (2.06-4.64), p<0.0001 / boys OR(95%CI): 2.18 (1.11-4.28), p<0.005]

O'Connor et al. 2014/ N. Ireland (CASE Study)	Exploration of the prevalence of DSH and related factors (exposure to the Troubles & relevant internet/social media pictures) in Northern Irish adolescents	Community-based adolescents 11-16 years; n= 3,526; RR: 80%	SBV was a risk factor for SI in both genders (The lifetime prevalence of SBV was strongly associated with SI, in both boys and girls): [girls OR(95%CI): 2.09 (1.59-2.73), p=0.0001 / boys OR(95%CI): 2.24(1.25-4.01), p=0.007].

Thomas et al. 2017/ Australia (2^nd^Australian & Adolescent Survey of Mental Health Wellbeing-Young Minds Matter Survey)	Exploration of the association between mental health, including self-harming & suicidal behaviour, and the three classes of bullying.	Community-based adolescents 11-17 years; n= 2967; RR: 89%	The risk factor for self-harming behaviour in those self-reported as bullying victims was higher compared to uninvolved participants [OR(95%CI):7.32(5.15-10.40)], adjusted score (gender, age). The mediating effect of DS on the association between SBV and SI was not assessed.

Fisher et al. 2012/England & Wales, UK (Environmental Risk Study)	Exploration of the association between the frequency of bullying victimization and occurrence of self-harm in early adolescence, as well as identifying which bullied children are at highest risk of self-harm.	Community-based twins 5-12 years; n= 2,232; RR:NR	Exposure to frequent SBV before the age of 12 years was a risk factor for SI at 12 years [RRisk(95%CI): 3.53 (2.10-5.93)- reports from mother]
			[RRisk (95% CI): 3.33 (1.91-5.82)-reports from child]
			Among the 62 children who engaged in self-harm, 35 (56%) had been victimized by bullying, while 42.8% of the self-harm cases could have been prevented [95% CI (23.1%-57.5%)] if SBV could be eliminated (other factors remaining constant). More than 90% of the children exposed to SBV did not engage in SI. Those who did were significantly more likely to (a) have a family member who attempted/completed suicide, (b) have been physically abused by an adult, and (c) exhibit conduct disorder, borderline personality characteristics, depressive, and psychotic symptoms.

Garish &Wilson 2015/ New Zealand	Investigation of the prevalence and correlates of NSSI in adolescents, including school bullying victimization.	Community-based adolescents 15-16 years (10^th^ grade); n= 830; RR: 60%	SBV was a significant predictor of NSSI Cross-sectional association between Lifetime NSSI & SBV: r= 0.31; p<0.01; Cross-sectional association between NSSI in the previous 3-8 months & SBV: r= 0.21, p<0.01)

Giletta et al. 2015/ China	Investigation of the predictive effect of school bullying victimization on latent trajectories of suicide ideation and NSSI in adolescents.	Community-based adolescents 15-16 years (10^th^ grade); n= 565; RR: 90.5%	Overt & relational SBV significantly predicted NNSI after controlling for gender & depressive symptoms, irrespective of the frequency of these behaviours. SBV differentiated the low (none or very few episodes) from the high frequency [OR(95%CI): 2.19 (1.42-3.39), the moderate from the low [OR (95%CI): 1.23(0.80-1.89)], and the moderate from the high NSSI group [1.02(0.98-1.07) ], independent of depressive symptoms. SBV differentiated the low (none or very few episodes) from the high frequency [OR(95%CI): 1.71 (1.19-2.47), the moderate from the low [OR (95%CI): 0.99(0.68-1.42)], and the moderate from the high NSSI group [1.74(01.18-2.56) ], independent of depressive symptoms. Adolescents in the high frequency NSSI group had more severe SBV scores than adolescents in the low and moderate frequency group (dose-response effect) (p <0.01).

Heilbron & Prinstein 2010/USA	Exploration of whether overt and relational peer victimization predicts suicidal ideation and NSSI, both concurrently and longitudinally.	Community-based adolescents 11-15 years; n= 493; RR: 73%-84%	A univariate association was reported between overt SBV and NSSI, with boys reporting NSSI being more frequently bullied compared to those who did not report NSSI (MANCONA; p<0.05), while girls who reported NSSI were less frequently victims of overt SBV (MANCONA; p<0.05). However, there was no main effect of the SBV on the prediction of NSSI when controlled for depressive symptoms.

Lereya et al. 2015/ USA & UK (Avon Longitudinal Study of Parents & Children; Great Smoky Mountains Study)	Exploration of the effects of maltreatment and bullying victimization on mental health status (i.e., self-harm, suicidality, and depressive symptoms) in adolescents.	Community-based adolescents 13-17 years; n= 5.446; RR: 78.5%	Children who were bullied by peers were significantly more likely to report SI compared to those who were not bullied [ALSPAC Cohort: OR (95% CI): 1.7 (1.4-2.2), p<0.0001& GSMS Cohort:; OR (95% CI): 3.0 (1.2-7.7), p=0.002] The experience of SBV has similar, and in some cases even worse, long-term adverse effects on the mental health of young adults compared to abuse by parents.

Lereya et al. 2013/ UK (Avon Longitudinal Study of Parents & Children)	Exploration of the hypothesis that school bullying victimization/SBV between the age of 7 and 10 is directly associated with self-injurious behaviour in late adolescence (16-17 years old).	Community-based adolescents 16-17 years; n= 4,810; RR: 77%	After controlling for all potential confounders SBV between the age of 7 and 10 was found to be associated with a greater risk of SI in late adolescence, based on reports from the child [OR(95% CI): 1.78(1.29-2.46)], the mother [OR(95%):CI 1.70 (1.27-2.28)] and the teacher [OR95%CI: 4.57 (1.66-12.54)]. The association between SBV and SI was partially mediated by depressive symptoms (B=0.21; SE=0.036 p<0.0001), since path analysis showed that SBV indirectly increased the risk of SI through the development of depressive symptoms (B=0.07; SE=0.02; p<0.0001).

SBV: school bullying victimization; NSSI: nonsuicidal self-injury; RR: response rate; n: number of participants; NM: not mentioned; NA: not assessed; SI: self-injury; SB: suicidal behaviour; and RRisk: relative risk.

**Table 2:** Presentation of the definitions and measures of self-injury, nonsuicidal self-injury and bullying victimization across studies.

**AUTHORS & YEAR/** **STUDY ACRONYM**	**TIME PERIOD ASSESSED FOR SELF-INJURY**	**DELIBERATE SELF-HARD (DSH), SUICIDALITY & BULLYING VICTIMIZATION DEFINITIONS USED IN THE STUDIES**	** SELF-INJURY, SUICIADAL BEHAVIOR & DEPRESSIVE SYMPTOMS MEASURES**	**BULLYING VICTIMIZATION MEASURES**
**Brunner et al. 2014 / the SEYLE study**	**Lifetime prevalence**	**BSH: **Intentional self- inflicted damage to the surface of an individual's body, which includes self-cutting, -burning, -biting, -hitting, and skin damage by other methods, regardless of suicidal intent.	**DSH: **6-item questionnaire (SEYLE study): (a) Have you ever intentionally cut your wrist, arms, or other area(s) of your body, or stuck sharp objects into your skin such as needles, pins, staples (not including tattoos, ear piercing, needles used for drugs, or body piercing)? (b) Have you ever intentionally burned yourself with a cigarette, lighter, or match? (c) Have you ever intentionally carved words, pictures, designs, or other markings into your skin or scratched yourself to the extent that scarring or bleeding occurred? (d) Have you ever intentionally prevented wounds from healing, or bit yourself to the extent that it broke skin? (e) Have you ever intentionally banged your head or punched yourself causing a bruise? (f) Have you ever intentionally hurt yourself in any of the abovementioned ways so that it led to hospitalization or injury severe enough to require medical treatment? **DS**: BDI	**Bullying victimization: **a single yes/no question

**Brunstein Klomek et al. 2016/ the SEYLE study**	**Lifetime prevalence**	**DSH:** Intentional self-inflicted damage to the surface of an individual's body, which includes self-cutting, -burning, -biting, -hitting, and skin damage by other methods, regardless of suicidal intent.	**DSH**: 6-item questionnaire about intentional self- inflicted damage of the surface of an individual's body by self-cutting, -burning, -hitting, -biting, and skin damage by other methods. (SEYLE: deliberate self-harm inventory-WSII) **DS:** BDI	**Physical/verbal/relational bullying victimization:** Ten yes/no questions about the three different types of victimization (e.g., “others pushed, hit or kicked you”; “others called you names”, “others spread rumours about you” & their frequency (occasional/ repetitive)

**Claes et al. 2015**	**Lifetime prevalence**	**NSSI: **Deliberate and direct injury of one's own body tissue without suicidal intent, such as scratching, cutting, hitting, and burning oneself.	**NSSI:** The NSSI subscale of the Self-Harm Inventory (SHI), comprising 22 yes/no items about the participant's intentional engagement in the described behaviour (cutting, burning, hitting, scratching, and head-banging). **DS: **Depressive Mood List-6 items	**Overt/relational bullying victimization:** Five items from the Olweus Bully/Victim self-reported questionnaire about direct bullying victimization, e.g., “How often were you left out of things, excluded, or ignored?” *Bullying victimization was defined as follows: being a victim of aggressive behaviour or intentional harm by others which is performed repeatedly over time and which involves an imbalance in power.*

**Elgar et al. 2014**	**Experiences during the past 12 months/30 days**	Not described	**DSH, Suicidal thoughts & attempts, DS: **Not stated how these variables were measured (depressive symptoms, self-harm, and suicid**e** attempt in the previous 12 months and suicidal thoughts in the previous 30 days).	**Overt/relational bullying victimization: **Four items about the frequency of face-to-face bullying victimization (being picked on, made fun of, called names, and hit or pushed by other students) from the Bullying and Victimization subscales of the University of Illinois Aggression Scales.

**Espelage & Holt 2013**	**Experiences during the past 6 months**	**DSH: **A broad definition of self-injurious behaviour including suicidal intent.	**DSH: **Two items from the Youth Self-report assessing students ‘suicidal ideation and self-injury history were combined into one composite variable. Each statement referred to the past 6 months and had the response alternatives: True and False: (1) “I deliberately try to hurt or kill myself;” or (2) “I think about killing myself. **DS:** Youth Self-report Anxiety & Depression scale-13 items.	**Overt/relational bullying victimization:** Four items about the frequency of face-to-face bullying victimization (being picked on, made fun of, called names, and hit or pushed by other students) from the Bullying and Victimization subscales of the University of Illinois Aggression Scales.

**Fisher et al. 2012**	**Lifetime prevalence**	**DSH: **Cutting and biting arms, pulling out clumps of hair, banging head against walls, and attempted suicide by strangulation.	**DSH:** A single question to the mothers of the twins if they had deliberately hurt themselves. **DS: C**DI	**Bullying victimization**: Assessed according to the following definition: **“**someone is being bullied when another child says mean and hurtful things, makes fun, or calls a person mean and hurtful names; completely ignores or excludes someone from their group of friends or leaves them out of things on purpose; hits, kicks, or shoves a person, or locks them in a room; tells lies or spreads rumours about them; or does other hurtful things; when these actions take place often/frequently and it is difficult for the person being bullied to stop it from happening”.

**Ford et al. 2017 **	**Experiences during the past 12 months**	**DSH: **Cutting oneself, overdosing on pills, or burning oneself	**DSH:** A single question about deliberate self-hurting (yes/no). **DS: **SMFQ scale	**Bullying victimization: **Assessed according to the following definition: For the next questions, please think about things that might have happened to you at school (or out of school). Include texts, Facebook, etc. as well as face-to-face contact. Do not include things that happened with your close family members (such as brothers & sisters).

**Garish &Wilson, 2015**	**Lifetime prevalence**	**NSSI**: Intentional, culturally unacceptable, self-performed, immediate, and direct destruction of bodily tissue that is of low-lethality and the absence of overdose, self-poisoning, and suicidal intent.	**NSSI**: Deliberate Self-Harm Inventory-Short form (WSII-s), precluding suicidal intent, regarding low lethality behaviours. **DS:** Zung SDS	**Bullying victimization:** Questions from section D of the Peer Relations Questionnaire assessing six different types and frequency of bullying victimization.

**Garish & Wilson, 2010**	**Lifetime prevalence**	**DSH:** Deliberate, nonfatal behavio**u**rs, intended to cause self-harm, including one or more of the following behaviours (self-cutting, jumping from a height, or ingestion of a substance or drugs in excess or a nondigestible substance or object	**DSH:** One item about deliberate self-harm (DeLeo & Heller's question) **DS**: Zung SDS	**Physical/verbal/relational bullying victimization**: Open-ended questions regarding the frequency of experiences (if/when/ how frequent) of physical, text/e-mail, verbal, or relational bullying victimization.

**Giletta et al. 2015**	**Experiences during the past 3 months**	**NNSI: **Direct and deliberate **self-**damage **to** one's body tissue without intention to die.	**NSSI:** 5-ite**m** questionnaire, each item referring to a specific NSSI method (i.e., cut/carved skin, burned skin, hit self, bit self, or scrapped skin to draw blood without intention to die). Frequency was also assessed. **DS: **CES-D scale	**Overt/relational bullying victimization:** A sociometric peer nomination procedure was used. Each adolescent was presented with a roster of all classmates and asked to identify those who they thought were victims of (i) overt/physical victimization (“Who gets beat up, picked on, or teased by bullies?”, “Who gets threatened or hit by others, or has mean things said to them?”) & (ii) relational victimization (“Who gets left out of activities or ignored by others because one of their friends is mad at them?”, “Who gets gossiped about or has rumours told about them behind their back?”**)**

**Giletta et al. 2012**	**Experiences during the past 6-12 months**	**NSSI:** Socially unacceptable, direct, deliberate destruction of one's own body tissue without suicidal intention.	**NSSI:** 6-item scale asking how frequently one intentionally engaged in several types of self-injurious behaviour (i.e., cut/carved skin, burned skin, hit self, bit self, scraping skin to draw blood, or inserting objects under skin or nails) without suicidal intent. **DS: **SMFQ scale	**Physical/verbal/relational bullying victimization: **3 items from the revised Olweus Bully/Victim Questionnaire about direct bullying victimization, i.e., “how often have you been victimized in the past 2 months at school (e.g., ‘How often were you beaten, kicked, or hit by peers?'), according to the following definition: ***“****We can say a student is a victim of bullying when another student or a group of peers says malicious or hurtful things to him. The same is true when a student is being hit, kicked, threatened, or excluded from the group. We call it “bullying” when these things happen frequently or regularly, and when it's difficult for the student being bullied to defend him or herself. It is NOT bullying when two or more students who are equally strong tease each other or fight with each other”*

**Gower & Borowsky, 2013/ the Minnesota Student Survey study**	**Experiences during the past 12 months**	**DSH: **Although the different types of self-directed violence were measured, the self-harm question did not include the intention or not to die.	**DSH:** One item asking whether participants had hurt themselves on purpose (e.g., cutting, burning, or bruising)	**Bullying victimization**: One single question about the frequency of different types of victimization: “During the last 30 days, how often have you been a victim of fun or teased by another student in a hurtful way or excluded from friends or activities?” Response options were ‘never', ‘once or twice', ‘about once a week', ‘several times a week', ‘every day'.

**Hay & Meldrum, 2010**	**Experiences during the past 12 months**	Not clearly described	**NSSI: ** how often did you purposely hurt yourself without wanting to die (i.e., cutting or burning yourself? **DS: **six-item scale	**Verbal/relational/physical bullying victimization**: a six-item scale: How often during the last 12 months were you: (i) the target of lies & rumours, and of attempts to get others to dislike you, (ii) called names, made fun of, or teased in a hurtful way, (iii) hit, kicked, or pushed by another student or physically threatened by other students, and (iv) picked on by others.

**Heilbron & Prinstein, 2010**	**Experiences during the past 12 months**	**NSSI: **Intentional, self-inf**l**icted body tissue damage, e.g., repetitive cutting, burning; conducted neither with suicidal intent nor in adherence to religious or cultural customs. **Bullycide: **The phenomenon of suicide due to bullying victimization.	**NSSI**: “In the past 12 months, have you ever harmed or hurt your body on purpose, such as cutting or burning your skin, or hitting yourself, without wanting to die?” **DS: **CDI scale	**Overt/relational bullying victimization: **Adolescents were asked to identify peers who were targets of the two forms of peer victimization in the school environment. Peer nomination items were used to index overt victimization (e.g., “Who gets threatened or hit by others or has mean things said to them?”) and relational victimization (i.e., “Who gets gossiped about or has rumours told about them behind their back?”

**Janter et al. 2015**	**Experiences during the past 12 months**	**NSSI: **Intentional self-inflicted damage to the surface of one's body without conscious suicidal intent, such as cutting or carving the skin, self-biting, or burning skin.	**NSSI: ** a single question clearly distinguishing NSSI from SB, asking about the intention (“without the intention to kill yourself”).	**Bullying victimization: **The victimization subscale from the Revised Olweus Bully/Victim Questionnaire (BVQ-R), including a clear definition of bullying victimization.

**Lereya et al. 2015/ the ALSPAC & GSMS studies**	**Lifetime prevalence**	**DSH:** An act with nonfatal outcome in which an individual deliberately hurts him/ herself, with or without the intention to die.	**DSH**: “Have you ever hurt yourself on purpose in any way (e.g., by taking an overdose of pills or by cutting yourself)?” (CIS-R questions) **DS**: CIS-R test	**Overt **(theft, threats, blackmail, physical violence, nasty names) & **Relational** (social exclusion, spreading lies or rumours, coercive behaviour, deliberately spoiling games) bullying victimization:
				(i) Modified version of the Bullying and FriendshipInterview Schedule **(ALSPAC study)**
				(ii) Child & Adolescent Psychiatric Assessment (CAPA) **(GSMS study)**

**Lereya et al. 2013/ the ALSPAC study**	**Lifetime prevalence**	**DSH**: An act with **a **nonfatal outcome in which an individual deliberately hurts him- or herself with or without the intention to die.	**DSH **: ”Have you ever hurt yourself on purpose in any way (e.g., by taking an overdose of pills or by cutting yourself)?” Those who responded positively were then asked about the frequency and the way they had hurt themselves. **DS: **SMFQ scale	**Overt/ relational bullying victimization: **Modified version of the Bullying and Friendship Interview Schedule; 5 questions on overt bullying: personal belongings taken; threatened or blackmailed; hit or beaten up; tricked in a nasty way; called bad/nasty names & 4 questions on relational bullying: exclusion to upset the child; pressure to do things she/he did not want to do; lies or nasty things said about him/her; and games spoiled.

**McMahon et al. 2010/ the CASE study**	**Lifetime prevalence**	**DSH**: An act with **a** nonfatal outcome in which an individual deliberately did one or more of the following: initiated behaviour (for example, self-cutting or jumping from a height), by which they intended to cause self-harm; ingested a substance in excess of the prescribed or generally recognized therapeutic dose; ingested a recreational or illicit drug that was regarded as self-harm; or ingested a noningestible substance or object.	**DSH: **“Have you ever tried to…according to the definition used”. Additionally, the participants were asked to describe the method(s) used to harm themselves in their own words. **SB: **No direct measurement **DS: **HADS	**Bullying victimization: **A single question about being bullied at school: “Have you ever been bullied at school?” including the timing of the event (more than a year ago or within the past year).

**Noble et al.2011**	**Lifetime prevalence**	**NSSI**: Deliberate, self-inflicted destruction of body tissue resulting in immediate damage, without suicidal intent and for reasons not socially sanctioned. This definition does not include suicidal or accidental injury, nor does it include eating disorders and substance abuse, which do not result in immediate tissue damage. The definition of NSSI also excludes body modification, such as tattooing and piercing, as these can be considered socially sanctioned behaviours. The most commonly reported NSSI behaviours include cutting, burning, scratching, and hitting oneself to cause bruising.	**NSSI**: “Have you ever physically hurt yourself on purpose**?”** If the students responded that they had deliberately hurt themselves in the past, they had to report the way they did it. **SB**: follow-up questions about the self-injurious behaviour, including a question asking if they had hurt themselves with intent to die.	**Bullying victimization**: A single yes/no question: “During the past 12 months, has someone bullied you on school property?”.

**O'Connor et al. 2014: the CASE study**	**Lifetime prevalence**	**DSH: **An act with a nonfatal outcome in which individuals deliberately did one or more of the following: initiated behaviour (e.g., self-cutting, jumping from a height), by which they intended to cause self-harm; ingested a substance in excess compared to the prescribed or generally recognized therapeutic dose; ingested a recreational or illicit drug in an act that the person regarded as self-harm; or ingested a noningestible substance or object.	**DSH:** “Have you ever deliberately taken an overdose (e.g., pills or other medication) or tried to harm yourself in some other way (such as cut yourself)?” Respondents were also asked to provide a description of the act and its consequences and to justify the motive behind the act **SB: **not directly measured **DS: **HADS	**Bullying victimization:** A single yes/no question about lifetime prevalence of being bullied at school: “Have you ever been bullied at school”?

**O'Connor et al. 2009/ the CASE study**	**Lifetime prevalence**	**DSH: **An act with a nonfatal outcome in which individuals deliberately did one or more of the following: initiated behaviour (e.g., self-cutting or jumping from a height), by which they intended to cause self-harm; ingested a substance in excess of the prescribed or generally recognized therapeutic dose; ingested a recreational or illicit drug that was regarded as self-harm; or ingested a noningestible substance or object.	**DSH**:“Have you ever deliberately taken an overdose (e.g., pills or other medication) or tried to harm yourself in some other way (such as cut yourself)?” Respondents were also asked to provide a description of the act and its consequences and to justify the motive behind the act **SB: **not directly measured **DS: **HADS	**Bullying victimization:** A single yes/no question about lifetime prevalence of being bullied at school: “Have you ever been bullied at school”?

**Thomas et al. 2017/ 2** ^**nd**^ ** Australian Child & Adolescent Survey of Mental Health& Wellbeing (Young Minds Matter Survey)**	Experiences during the past 12 months	**DSH: **An act with a nonfatal outcome in which individuals deliberately harmed or injured themselves without intending to end their own life during the past 12 months.	**DSH: **“Have you deliberately harmed or injured yourself without intending to end your own life during the past 12 months (never/ self-harmed in the past year/prefer not to say )” **DS:** Diagnostic Interview Schedule for Children, 4 (DISC-IV)	**SBV:** Single global item “In the past 12 months, how often were you bullied or cyberbullied by another person or group of young people?” & 10-item scale adapted from Olweus Bully-Victim Questionnaire (for once or more often respondents), Cyber Friendly Schools program

**DSH: **deliberate self-harm irrespective of suicide intent;** DS: **depressive symptoms;** NSSI: **nonsuicidal self-injury;** SB: **suicidal behaviour; **SMFQ:** Short Mood & Feelings Questionnaire; **HADS:** Hospital Anxiety & Depression Scale; BDI: Beck Depression Inventory; **CIS-R test: **Clinical Interview Schedule-Revised test; **CDI:** Children's Depression Inventory; **Zung SDS: **Zung Self-rating Depression Scale**; CES-D scale: **Center for Epidemiological Studies-Depression.

**Table 3:** Methodological characteristics of the studies in the present systematic review.

**Authors & Year/Country**	**Study Design**	**Sample**	**Measurement of the Main Variables (SBV/SI-NSSI)**	**Confounding Factors Assessed**	**Limitations**	**NOKC /CASP** **Quality** **Assessment** **(low, moderate,** **high)**
Brunner et al. 2014/ 11 European countries	Cross-sectional, comparative, correlational study	Random sample; mean age: 14.9 years; n= 12,068	Self-reported questionnaires: open-ended questions & psychometric scales	Demographic data; income; family type; immigrant status; religiosity; psychopathology; suicidality; anxiety & depressive symptoms; substance abuse; parenting; social relationship problems & loneliness; quality of parenting & communication with parents; impulsivity	Self-reported data. No longitudinal data, thus the study cannot provide information about causality; no triangulation of data with teachers/ parents/peer nomination reports	Moderate quality

Brunstein Klomek et al. 2016/ 10 European countries	Cross-sectional, correlational study	Random sample; mean age: 14.9 years; n= 11,110	Self-reported questionnaires: open-ended questions & psychometric scales	Demographic data; income; family type; immigrant status; religiosity; psychopathology; suicidality; anxiety & depressive symptoms; substance abuse; parenting & support; social relationship problems, peer support & loneliness; quality of communication with parents; impulsivity; prosocial behaviour	Self-reported data; the cross-sectional nature of the study does not allow assumptions on causality; no triangulation of data with teachers/ parents/ peer nomination reports	Moderate quality

Claes et al. 2015/ Belgium and the Netherlands	Cross-sectional & correlational study	Convenience sample; mean age: 15.56 years; n= 785	Self- reported questionnaires, psychometric scales	Depressive symptoms; perceived parental support; age; gender; victimization.	Self-reported data; no triangulation of data with teacher/ parent/ peer nomination reports; only the presence/absence of NSSI was assessed; data were gathered at one point in time; no conclusions on causality; important confounders were not assessed, i.e., impulsivity, drug abuse, self-esteem, mental health problems	Moderate quality

Elgar et al. 2014/USA	Cross-sectional, observational & correlational study	Random sample; mean age 15.0; n=18,834	Anonymous, self-reported, electronically distributed questionnaires: psychometric scales & open-ended questions	Cyber bullying; victimization; anxiety & depressive symptoms; self-harm & suicidal behaviour; physical fighting & vandalizing; substance misuse (alcohol & legal & illegal drugs); family communication/ support; household income; age; gender	Self-reported data; no triangulation of data with teacher/ parent/ peer nomination reports; cross-sectional design, thus no conclusions on causality	Moderate quality

Espelage & Holt 2013/ USA	Cross-sectional study	Random sample; median age 12.3 (range: 10-13); n=661	Anonymous, self-reported questionnaires, Psychometric scales	Anxiety & depressive symptoms; delinquency; suicidal ideation; gender; grade; race	Self-reported data; no triangulation of data with teachers/ parents/ peer nomination reports; cross-sectional design, thus no conclusions on causality; important confounders were not included, e.g. substance use.	Moderate quality

Ford et al. 2017/Australia	Cross-sectional study	Random sample; median age NR (range: 14-15); n=2304	Anonymous, self-reported questionnaires, psychometric scales, face-to-face interviews & computer-assisted interviews.	Gender, household type & income; language spoken in home; parents' education; Aborigin/ Torres Strait	Cross-sectional design, thus no conclusions on causality; important confounders were not assessed, i.e. substance misuse, self-esteem, impulsivity	High quality

Garish & Wlilson 2010/ New Zealand	Cross-sectional & correlational, exploratory study.	Convenience sample; mean age: 16.67 years; n=325	Anonymous, self-reported questionnaires: psychometric scales & open- ended questions	Depressive symptoms; alexithymia; gender	Self-reported data & recall bias; no triangulation of data with teacher/ parent/ peer nomination reports; cross-sectional design, thus no conclusions on causality; important confounders were not assessed, i.e. substance misuse, self-esteem, impulsivity; generalizability limited to adolescents of European origin with a high socioeconomic status.	Moderate quality

Giletta et al. 2012/ USA, Italy, The Netherlands	Cross-sectional & correlational study	Convenience sample; mean age=15.7 years; n=1,862	Anonymous, self-reported questionnaires: psychometric scales & open- ended questions	Age, gender, ethnicity and parents' educational level; depressive symptoms; family & peer related loneliness; peer preference (interpersonal stressors); substance use	Self-reported data; no triangulation of data with teachers/ parents/ peer nomination reports; cross-sectional design, thus no conclusions on causality; important confounders were not assessed, i.e. self-esteem, impulsivity; low response rate in the subgroups; convenience sample	Moderate quality

Gower & Borowsky 2013/ USA	Cross-sectional study	Convenience sample; mean age: NR; n=128,681	Self-reported questionnaires, psychometric scales	Age; gender; ethnicity; impulsivity; suicidality; personal & parental mental health problems & substance use; emotional distress; family conflict & running away; skipped school; negative self-concept; religious activities; supportive social network; conduct problems; depressive and anxiety symptoms; emotional & physical domestic violence; physical & sexual abuse; witness to domestic violence; parent connectedness; academic performance; family structure & income; residency	Self-reported data; no triangulation of data with teacher/ parent/ peer nomination reports; no causality	Moderate quality

Hay & Meldrum, 2010/ USA	Cross-sectional, noncomparative, correlational study	Convenience sample; mean age: 15 years; n=424	Self-reported questionnaires: open-ended questions & psychometric scales	Age; gender; ethnicity/origin; family type; school performance; impulsivity; authoritative parenting	No triangulation of data with teacher/ parent/ peer nomination reports; no causality; convenience sample; no assessment of mental health variables as confounders	Moderate quality

Jantzer et al. 2015/ Germany	Cross-sectional, noncomparative, correlational study	Entire target population; mean age: 12.8 years; n= 647	Self-reported questionnaires: open-ended questions & psychometric scales	Age; gender; suicidal behaviour; grade; parental monitoring	No triangulation of data with teacher/ parent/ peer nomination reports; no causality; convenience sample; no assessment of important confounders, e.g. impulsivity; self-esteem, etc.	Low quality

McMahon et al. 2010/Ireland	Cross-sectional, noncomparative, correlational study	Random sample; mean age: 16 years; n= 3,881	Self-reported questionnaires: open-ended questions & psychometric scales	Anxiety; impulsivity; self-esteem; DSH of a friend/ family member; drug use; sexual abuse; friendship difficulties; fights with parents; dysfunctional school performance	No triangulation of data with teacher/ parent/ peer nomination reports; no causality assessment; no assessment of important confounders, e.g. substance use; no assessment of social support variables; exclusion of those who did not describe DSH behaviour (risk of underestimation of the prevalence)	Moderate quality

Noble et al. 2011/ USA	Cross-sectional, comparative study	Purposeful (matched groups) sample; mean age: 14.9 years; n= 1,276	Self-reported questionnaires: open-ended questions & psychometric scales	Perceived trust in school context (trust in students/teachers/administration/school counsellor) & safety (missed days due to feeling unsafe; carrying a weapon/ threatened/being bullied at school)	No triangulation of data with teacher/ parent/ peer nomination reports; no causality assessment; convenience sample; no assessment of mental health variables as confounders; social support variables were not included	Moderate quality

O'Connor et al. 2009/Scotland, UK	Cross-sectional, noncomparative, correlational study	Random sample; mean age: 15 years; n= 2,008	Self-reported questionnaires: open-ended questions & psychometric scales	Anxiety & depression symptoms; impulsivity; self-esteem; DSH of a friend/ family member; drug use; sexual abuse; friendship difficulties; fights with parents; dysfunctional school performance	No triangulation of data with teacher/ parent/peer nomination reports; no causality assessment; no assessment of social support variables	Moderate quality

O'Connor et al. 2014/ Northern Ireland	Cross-sectional, noncomparative, correlational study	Random sample; mean age: 15 years; n= 3,596	Self-reported questionnaires: open-ended questions & psychometric scales	Anxiety; depression; impulsivity; self-esteem; DSH of a friend/ family member; drug/alcohol use; sexual/physical abuse; sexual orientation concerns; exercising; living with both parents; exposure to internet/TV DSH images; exposure to difficulties related to “The Troubles”	No triangulation of data with teacher/ parents/ peer nomination reports; no causality assessment; no assessment of social support variables	Moderate quality

Thomas et al. 2017/ Australia	Cross-sectional, correlational study	Random, nationally representative sample; mean age: 14.6 years; n=2967	Self-reported questionnaires: open-ended questions & psychometric scales Face-to-face structured interview for parents/carers	Age, gender	Cross-sectional design, thus no conclusions on causality; important confounders were not assessed, i.e. substance misuse, self-esteem, impulsivity, etc; over-representation of socially/income advantaged families	High quality

Fisher et al. 2012/ UK	Longitudinal birth cohort, comparative study	Birth cohort sample; mean age*: *NR; n= 2,232	Clinical interviews of mothers/children /teachers: open-ended questions & psychometric scales	Exposure to physical/ sexual maltreatment; anxiety symptoms; depressive symptoms; withdrawn, aggressive & delinquent behaviour; IQ;	The small number of children who engaged in self-injurious behaviour led to biased estimations about the size of the association between the main variables; no inclusion of important confounders, i.e. substance use; parenting & social support variables were not included	High quality

Garisch & Wilson, 2015/New Zealand	Prospective study with measurement at two time points	Random sample; mean age: 16.34(T1) -16.45(T2) years; n=830	Self-report questionnaires	Gender; anxiety & depressive symptoms; self-esteem; alexithymia; adaptive emotional response; resilience; impulsivity; physical & sexual abuse history; substance abuse; sexuality concerns; mindfulness	Moderate internal consistency & test-rest reliability of the instruments applied	High quality

Giletta et al. 2015/China	Prospective cohort comparative study	Random sample; 10^th^ grade (mean age: 16 years); n=565	Self-reported questionnaires & peer nominated data	Gender; suicidal ideation; depressive symptoms; stressful peer experiences/ type and quality of friendships; friend support	Peer nominated data regarding overt and relational school bullying victimization, excluding subjective experiences; the low NSSI/ SI trajectory group included those reporting no or very few episodes, thus the degree to which school bullying victimization differentiated those who engaged in SITB from those who did not at all was not clearly reported; no assessment of substance use as a confounder	High quality

Heilbron & Prinstein 2010/ USA	Longitudinal, population-based comparative cohort study	Random sample; mean age: 12.6 years; n= 493	Clinical interviews with students and peers	Peer status/popularity; depressive symptoms; gender; suicidal ideation	Only partially ethnically diverse sample; no inclusion of important confounders in the analysis/study design, i.e., substance abuse, impulsivity, suicidal behaviour (suicide attempts & plans); parenting/ social support was not assessed; small group sizes in the internal comparisons	High quality

Lereya et al. 2015/ USA & UK	Comparative study of longitudinal birth cohort & population-based data.	Cohort sample; mean age: NR; n= 5,446	Self-reported postal questionnaires about self-harm variables from the adolescents at the age of 16-17 years; face-to-face interviews with the children at the age of 8 & 10 years/mothers/ teachers about predictor variables	Gender; ethnicity; parents' educational level & marital status; parental mental health problems; parental stress; family conflict; preschool maladaptive parenting (hitting, shouting, hostility); conduct problems; hyperactivity; depressive and anxiety symptoms; emotional & physical domestic violence; borderline personality disorder symptoms; sexual abuse	Self-reported data & recall bias; face-to-face interviews & embarrassment bias	High quality

Lereya et al. 2013/ UK	Longitudinal birth cohort, comparative study	Birth cohort sample; mean age: NR; n= 4,810	Self-reported postal questionnaires about self-harm variables from the adolescents at the age of 16-17 years; face-to-face interviews with the children at the age of 8 & 10 years/mothers/ teachers about predictor variables.	Gender; preschool maladaptive parenting (hitting, shouting, hostility); conduct problems; hyperactivity; depressive symptoms; emotional & physical domestic violence; borderline personality disorder symptoms	Self-reported self-harm & recall bias; no assessment of social support; face-to-face interviews & embarrassment bias.	High quality

SBV: school bullying victimization; NSSI: non-suicidal self-injury; SI: self-injury.

**Table 4:** The appraisal of the methodological integrity of the studies in the present systematic review performed by two independent researchers in accordance with the NOKC instrument assessment criteria (n=13) for the cross-sectional studies (both analytic comparative and noncomparative studies), and the CASP instrument criteria for the cohort studies.

	**Independent Assessment #1**														**Independent Assessment #2**												
	**NOKC INSTRUMENT**														**NOKC INSTRUMENT**												
	**Methodological Integrity Criteria**														**Methodological Integrity Criteria**												
	CR1	CR2	CR3	CR4	CR5	CR6	CR7	CR8	CR9	CR 10	CR 11	CR12	CR 13		CR1	CR2	CR3	CR4	CR5	CR 6	CR7	CR8	CR9	CR10	CR11	CR12	CR13
**Authors and year of publication**																											
Brunner et al. 2014	V	V	NA	NA	NR	PV	V	V	V	NA	NA	V	V		V	V	NA	NA	V	NV	V	V	V	NA	NA	V	V
Brunstein Klomek et al. 2016	V	V	NA	NA	NR	PV	V	V	V	NA	NA	V	V		V	V	NA	NA	NV	V	V	V	V	NA	NA	V	NR
Claes et al. 2015	V	V	NA	NA	V	V	V	V	V	NA	NA	PV	V		V	V	NA	NA	V	V	V	V	V	NA	NA	PV	V
Elgar et al. 2014	V	V	NA	NA	V	V	V	V	V	NA	NA	V	V		V	V	NA	NA	V	V	V	V	V	NA	NA	V	V
Espelage & Holt 2013	V	V	NA	NA	V	V	V	PV	V	NA	NA	V	V		V	V	NA	NA	V	V	V	PV	V	NA	NA	V	V
Ford et al. 2013	V	V	V	NR	V	PV	V	V	V	V	V	PV	V		V	V	V	NR	V	PV	V	V	V	V	V	V	V
Garich & Wilson 2010	V	V	NA	NA	NV	V	V	V	V	NA	NA	PV	V		V	V	NA	NA	NV	V	V	V	V	NA	NA	PV	V
Giletta et al. 2012	V	V	NA	NA	V	NV	NV	V	V	NA	NA	PV	V		V	V	NA	NA	V	NV	NV	V	V	NA	NA	PV	V
Gower & Borowsky 2013	V	V	NA	NA	NV	NR	V	V	V	NA	NA	V	V		V	V	NA	NA	NV	NR	V	V	V	NA	NA	V	V
Hay & Meldrum 2010	V	PV	NA	NA	NR	V	V	V	V	NA	NA	V	V		V	PV	NA	NA	NR	V	V	V	V	NA	NA	V	V
Jantzer et al. 2015	V	NR	NA	NA	NR	NR	V	V	V	NA	NA	PV	V		V	NR	NA	NA	NR	NR	V	V	V	NA	NA	PV	V
McMahon et al. 2010	V	V	NA	NA	PV	V	V	V	V	NA	NA	V	V		V	V	NA	NA	PV	V	V	V	V	NA	NA	V	V
Noble et al. 2011	V	V	V	V	PV	NR	PV	V	V	V	V	NV	V		V	V	V	V	PV	NR	PV	V	V	V	V	NA	V
O'Connor et al. 2009	V	V	NA	NA	V	V	V	V	V	NA	NA	V	V		V	V	NA	NA	V	V	V	V	V	NA	NA	V	V
O'Connor et al. 2014	V	V	NA	NA	V	V	V	V	V	NA	NA	V	V		V	V	NA	NA	V	V	V	V	V	NA	NA	V	V
Thomas et al. 2017	V	V	V	NV	NV	V	V	V	V	V	V	NV	V		V	V	V	NA	V	V	V	V	V	V	V	NV	V

	**Independent Assessment #1**														**Independent Assessment #1**												
	**CASP INSTRUMENT**														**CASP INSTRUMENT**												
	**Methodological Integrity Criteria**														**Methodological Integrity Criteria**												
CASP	1	2	3	4	5	6	7	8	9	10	11	12	13	1	2	3	4	5	6	7	8	9	10	11	12	13	
Fisher et al. 2012	V	V	V	V	V	V	V	V	V	V	V	V	V	V	V	V	V	V	V	V	V	V	V	V	V	V	
Garisch & Wilson 2015	V	V	V	V	NR	V	V	V	V	V	NR	V	V	V	V	PV	PV	V	NV	V	PV	PV	V	V	V	V	
Giletta et al. 2015	V	V	V	V	NR	V	V	V	V	V	V	V	V	V	V	PV	V	V	PV	PV	V	V	V	V	V	V	
Heilbron & Prinstein 2010	V	V	V	V	NR	V	V	V	V	V	NR	NR	V	V	V	V	PV	PV	PV	V	PV	PV	PV	V	V	V	
Lereya et al. 2015	V	V	V	V	V	V	V	V	V	V	V	V	V	V	V	V	V	V	V	V	V	V	V	V	V	V	
Lereya et al. 2013	V	V	V	V	V	V	V	V	V	V	V	V	V	V	V	V	V	V	V	V	V	V	V	V	V	V	

V: valid

NV: not valid

NR: not reported

PV: partially valid

NA: not applicable

CR: criterion of rigour.

## Data Availability

All data and materials are available from Dr. Maria Karanikola.
